# Transcriptional Profiling of Mouse Uterus at Pre-Implantation Stage under VEGF Repression

**DOI:** 10.1371/journal.pone.0057287

**Published:** 2013-02-28

**Authors:** Yan Ji, Xiaodan Lu, Qingping Zhong, Peng Liu, Yao An, Yuntao Zhang, Shujie Zhang, Ruirui Jia, Isaias G. Tesfamariam, Abraha G. Kahsay, Luqing Zhang, Wensheng Zhu, Yaowu Zheng

**Affiliations:** 1 Transgenic Research Center, School of Life Sciences, Northeast Normal University, Changchun, China; 2 KLAS and School of Mathematics and Statistics, Northeast Normal University, Changchun, China; University of Texas-Houston Medical School, United States of America

## Abstract

Uterus development during pre-implantation stage affects implantation process and embryo growth. Aberrant uterus development is associated with many human reproductive diseases. Among the factors regulating uterus development, vascular remodeling promoters are critical for uterus function and fertility. Vascular endothelial growth factor (VEGF), as one of the major members, has been found to be important in endothelial cell growth and blood vessel development, as well as in non-endothelial cells. VEGF mediation in reproduction has been broadly studied, but VEGF-induced transcriptional machinery during implantation window has not been systematically studied. In this study, a genetically repressed VEGF mouse model was used to analyze uterus transcriptome at gestation 2.5 (G2.5) by Solexa/Illumina’s digital gene expression (DGE) system. A number of 831 uterus-specific and 2398 VEGF-regulated genes were identified. Gene ontology (GO) analysis indicated that genes actively involved in uterus development were members of collagen biosynthesis, cell proliferation and cell apoptosis. Uterus-specific genes were enriched in activities of phosphatidyl inositol phosphate kinase, histone H3-K36 demethylation and protein acetylation. Among VEGF-regulated genes, up-regulated were associated with RNA polymerase III activity while down-regulated were strongly related with muscle development. Comparable numbers of antisense transcripts were identified. Expression levels of the antisense transcripts were found tightly correlated with their sense expression levels, an indication of possibly non-specific transcripts generated around the active promoters and enhancers. The antisense transcripts with exceptionally high or low expression levels and the antisense transcripts under VEGF regulation were also identified. These transcripts may be important candidates in regulation of uterus development. This study provides a global survey on genes and antisense transcripts regulated by VEGF in the pre-implantation stage. Results will contribute to further study the candidate genes and pathways in regulating implantation process and related diseases.

## Introduction

Infertility has been a serious global problem, especially in developed countries [1]. The World Health Organization estimates that 8–12% of all couples experience infertility worldwide and about 40 million couples undergo infertility in China. Of the pregnancies that are lost, 75% represents a failure of implantation. Implantation failure is a major factor in assisted reproduction [2]. Extensive prenatal mortality has been observed in farm animals [3]. Ruminants experience relatively high levels of pregnancy loss during the pre-implantation period. Prenatal death results in reducing litter size in pigs and prolific sheep. Most of these embryonic loss also happen in implantation stages [4]. Failure in implantation leads to an increased interval between births and economic loss of farm [3].

Implantation is a critical process where conceptus comes close to and initiates development at endometrial epithelium surface [5]. A complex network of signaling molecules, adhesive factors and functional effectors may be involved during the process. Signaling molecules such as transforming growth factor betas (TGFβs), integrins and VEGF have been documented but the complete picture is still missing [6], [7], [8]. VEGF is essential for embryonic vasculogenesis and angiogenesis, as well as tumor angiogenesis [9], [10], [11]. Proper level of VEGF expression is required for implantation [12], [13].

High-throughput sequencing technology has been broadly used for transcriptome analysis [14]. Taking advantage of the Solexa/Illumina Genome Analyzer platform, we performed transcriptional profiling on the VEGF-normal (Dox+) and VEGF-repressed (Dox**−**) mouse uteri. The DGE tag profiling allows us to analyze gene expression level of these samples in full scale [15], [16], [17]. Based on the data, uterus-expressed genes, uterus-specific genes and VEGF-regulated genes were analyzed. Related signaling pathways were evaluated.

## Materials and Methods

### Ethics Statement

Animals were maintained in the animal facility following the guidelines of Laboratory Animal Resource Center of Northeast Normal University, originally developed and supervised by the China Council on Animal Care, and protocol approved by the Committee on Animal Research of Jilin Province. The mice were kept in pathogen-free animal facilities in Northeast Normal University, 12 h light/dark cycles and free access to food and water. Mice were anesthetized before sacrificing with 1% pelltobarbitalum natricum at the dose of 10 mg/kg.

### Animal and Tissue Collection

**Figure 1:**
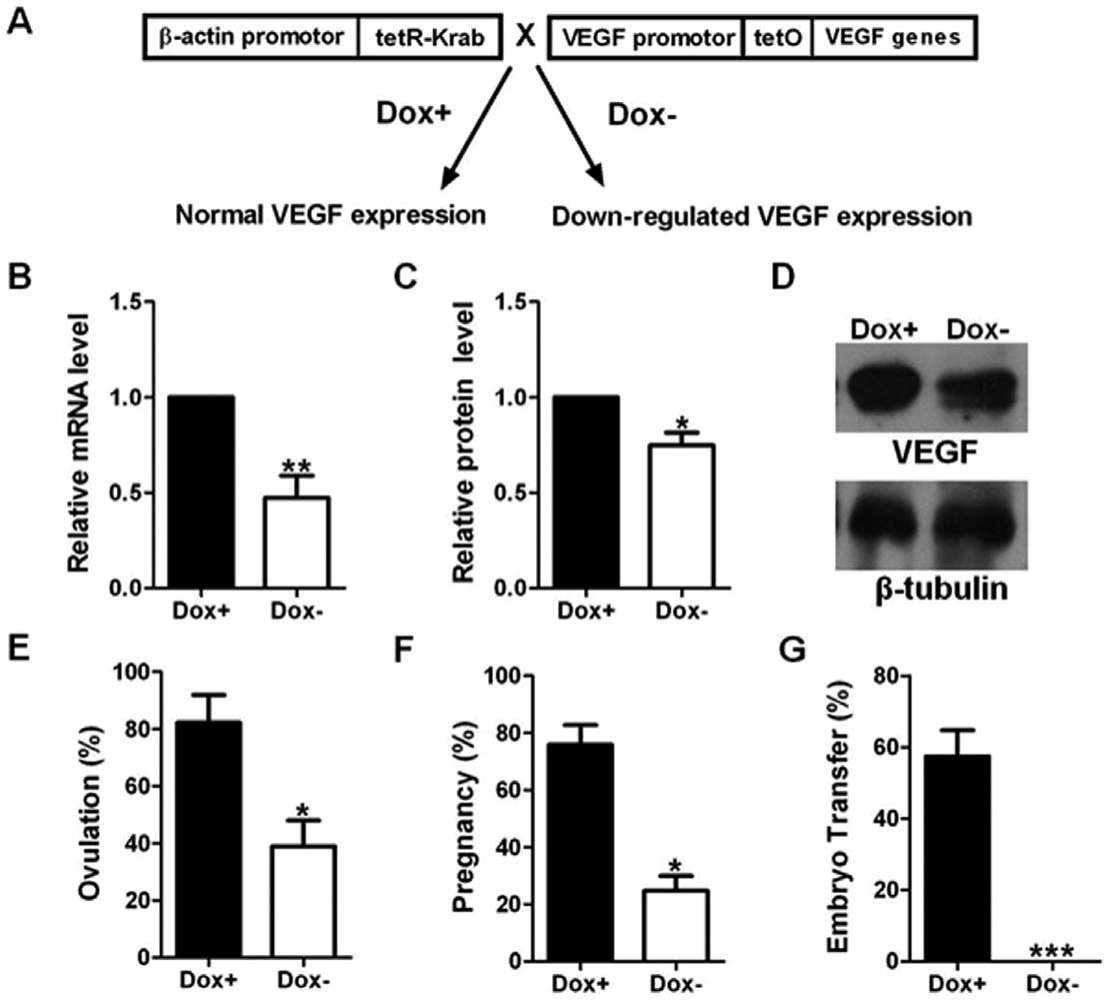
VEGF repression effect on ovulation, pregnancy and implantation rate. (**A**) Reversible repression system of VEGF gene expression. (**B**) Relative VEGF mRNA levels by qPCR (n = 5). (**C**, **D**) Protein expression by Western blot analysis and the quantification (n = 3). (**E**) Embryo collection at E3.5 (n = 45). (**F**) Embryo dissection at E9.5 (n = 11). (**G**) Embryo transfer experiment (n = 22). Dox+ represented mouse chow with 200 mg/kg doxycycline. Dox**−** represented regular mouse chow. Data are presented as mean ± SEM. *P<0.05, **P<0.005, ***P<0.001.

Double transgenic mice VEGF^tetO/tetO^/β-actin-tetR-Krab (βAK^tg/wt^) were generated as described previously [18], [19]. In brief, four copies of tet operator (tetO) sequences were inserted into the promoter region of VEGF by gene targeting (VEGF^tetO^). The transgenic mice carrying universal expression of tetR-Krab fusion protein were generated by pronuclei DNA injection (β-actin-tetR-Krab). By crossing two lines, VEGF expression was under control of tetracycline (Figure 1A). When tetracycline is absent (Dox**−**), tetR-Krab fusion protein binds to VEGF promoter region and blocks VEGF expression. All the embryos die at E10.5. When tetracycline is administered in the food (Dox+), tetR-Krab fusion protein binds to tetracycline and falls off VEGF promoter VEGF expression goes back to the normal, following Mendel inheritance. These mice were phenotypically normal. The propagation of double transgenic mice in this experiment, therefore, was maintained on doxycycline food (200 mg/kg), a tetracycline analogue. Doxycycline was removed at the age of 3–4 weeks and total RNA was isolated from littermates of Dox+ and Dox**−** uteri after 6 weeks. All the mice (Dox+ and Dox**−**) used were littermates and analyzed at the age of 9–10 weeks. Double transgenic females (VEGF^tetO/tetO^/βAK^tg/wt^) were set for timed mating with VEGF males (VEGF^tetO/tetO^). The day of detecting vaginal plugs was designated as G0.5. Animals were euthanized and tissues were collected at G2.5. Uterine samples from two groups Dox+ and Dox**−** were collected for RNA isolation.

### Ovulation, Implantation and Embryo Transfer Studies

Double transgenic females (VEGF^tetO/tetO^/βAK^tg/wt^) without doxycycline food (Dox**−**) were mated with VEGF male mice (VEGF^tetO/tetO^). Mice were genotyped by PCR tetO insertion region and presence of tetR as described previously [18]. VEGF^ tetO/tetO^ mice were phenotypically normal as wild type. Mice were euthanized and uteri were excised at G3.5 and G9.5. M2 solution (Sigma) was used to flush the G3.5 uteri and for blastocyst collection. Blastocysts were harvested and counted using an OLYMPUS DP72 scope. Ovulation of VEGF^ tetO/tetO^/βAK^tg/wt^ mating on doxycycline food (Dox+) was used as a positive control.

Due to the combination of problems in ovulation and implantation in lower pregnancy of Dox**−**, the implantation efficiency was evaluated by transferring wild type (VEGF^tetO/tetO^) blastocysts (E3.5) into pseudo-pregnant females of VEGF^tetO/tetO^/βAK^tg/wt^ with (Dox+) and without (Dox−) doxycycline food at G3.5. For the timed mating, vaginal plug was checked and designated as G0.5. Implantation site was confirmed at G9.5 by embryo dissection. Presence of developing embryo was considered as successful implantation and absence was considered as failure in implantation.

### Samples Preparation for High throughput RNA Tag Sequencing

Total RNA was isolated from VEGF repressed (Dox**−**) and control (Dox+) uteri. Further sample processes were performed by following manufacturer’s instructions and subjected to Solexa/Illumina sequencing. Total RNA was isolated from three uteri of Dox+ and Dox**−** at G2.5 using RNAiso™ Plus (TAKARA). The RNA was treated with RNase-free DNase to remove potential genomic DNA contamination. RNA integrity and concentration were confirmed by gel electrophoresis and Agilent 2100 Bioanalyzer (Agilent Technologies, Palo Alto, CA, USA). For RNA library construction and deep sequencing, 6 µg of total RNA from three individual Dox+ and Dox**−** uteri were pooled. All procedures were performed by following the manufacturer’s instruction.

### Sequencing Data Analysis

A reference tag database (Unigene) which included 22198 sequences from *Mus musculus* was used for tag alignment (http://hgdownload.cse.ucsc.edu/goldenPath/mm9/bigZips/refMrna.fa.gz). To produce total clean tags, adaptor only, low quality sequences and tags with copy number less than 2 were removed. The copy number corresponding to each gene was counted and standardized. Clean tags were calculated and normalized to Transcripts Per Million (TPM) for comparison between samples. Both sense and antisense sequence tags were collected and searched against the reference tags. The unambiguous tags were annotated. Raw data has been deposited to GEO database under submission number GSE43351.

### Analysis of Uterus-expressed Genes

Dox+ group, which was considered as wild type like group, was used to represent uterus-expressed genes. Genes with copy number ≥100 were considered as meaningfully expressed genes and were used for further analysis.

### Identification of Uterus-specific Genes

To identify uterus-specific genes, uterus-expressed genes were compared with those genes expressed in the brain, liver, and muscle respectively. Considering most genes were expressed at very low levels and may not represent significant biologic effectors, the cutoff of ≥100 greatly reduced gene numbers by more than 50%. The data cutoff of other tissues was also reset accordingly. The genes present only in uterus were considered as uterus-specific.

### Analysis of VEGF-regulated Gene

To compare differentially expressed genes between the two samples (Dox+ and Dox**−**), genes with combined copy number of Dox+ and Dox**−** ≥200 were selected. A statistical analysis of VEGF-regulated genes was performed according to the method described by Audic *et al* [20]. False discovery rate (FDR) was used to determine the threshold of P-value in multiple test and analysis. We used P-value <0.05, FDR <0.05 and the absolute value of log2^Ratio^ change >0.5 to set the cutoff of VEGF-regulated genes.

### GO Analysis and Signaling Pathway Analysis

We have used GOrilla [21], David [22], Gprofiler [23] and Funnet [24] to analyze biological processes (BP), cellular components (CC) and molecular functions (MF). GO annotation showed similar results and all the results were presented based on GOrilla analysis. Within the significant category, the enrichment was given by: 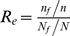 (Re = Enrichment), *n_f_* is the number of flagged proteins within the particular category, *n* is the total number of proteins within the same category, *N_f_* is the number of flagged proteins in the reference database list, *N* is the number of proteins in the gene reference database list.

Pathway analysis was based on the Kyoto Encyclopedia of Gene Genomics (KEGG) database [25]. Two-side Fisher’s exact test with multiple testing and χ^2^ test were used to classify the pathway category. Pathway categories that had a P-value <0.05 were selected.

FDR was used to correct the P-value. GO and KEGG analysis has satisfaction values of P<0.05 and FDR <0.05.

### Analysis of Antisense Transcripts

Dox+ and Dox**−** antisense transcripts with copy number ≥100 were analyzed.

### Validation of Gene Expression

Differential expression of VEGF-regulated genes was validated by quantitative real time PCR (qPCR). The RNA samples used for qPCR analysis were isolated from Dox+ and Dox**−** uteri at G2.5 according to previously described method [19]. For the first-strand cDNA synthesis, 1 µg total RNA was reversibly transcribed with RT kit (RNAiso™ Plus) from TAKARA. Primers were synthesized by Genewiz (Suzhou, China). Gene expression was assessed by qPCR using LightCycler 480 Sequence Detective System (Roche) and SYBR Green (TOYOBO). All PCR results were normalized to the expression level of 18S. Relative expression level was calculated with 2^−ΔΔCt^ value method. Data were presented as mean ± SEM of triplicate samples and repeats of 3 times. PCR primers were listed in Table S7.

### Statistical Analysis

Data were shown as mean ± SEM. Student’s t-test was used to calculate the statistical significance of experimental results. P-value <0.05 is considered statistically significant.

## Results

### VEGF Repression Caused Implantation Failure

A genetic mouse model of reversible VEGF repression was used in this study (Figure 1A) [18], [19], [26]. VEGF mRNA expression levels among different tissues have been determined by qPCR (data not shown). VEGF mRNA expression level was down-regulated by 54% (Figure 1B) and protein expression level by 33% (Figure 1C and 1D) in uterus after removing doxycycline (Dox**−**). VEGF repressed mice showed lower efficiency of pregnancy, about 24.7% of normal (Figure 1F), which was determined by embryo dissection at G9.5. In order to determine whether ovulation or implantation has caused the decrease in live birth, ovulation was determined by collecting embryos at E3.5 and the implantation efficiency was evaluated by transferring wild type blastocysts (E3.5) into pseudo-pregnant females with and without doxycycline food (Dox+ and Dox**−**) at G3.5. VEGF repression has caused a decrease in both ovulation and implantation. Ovulation was decreased by 38.9% (Figure 1E). No implantation was detected in Dox**−** while control (Dox+) showed 58% of implantation efficiency (Figure 1G).

### Tag Mapping and Data Evaluation

Results were summarized in Table S1. Over 6 million raw tags were obtained from each sample. Saturation analysis of the libraries was shown in Figure S1. The number of total tags obtained was much higher than required to saturate. After filtering out low quality tags, adaptor only tags and tags appeared only once, over 97% of tags were retained from both Dox+ and Dox**−** tag libraries. These tags were defined clean tags for further analysis. Total mapped tags were 86.96% and 85.34%, equal to distinct tags 62.89% and 67.80% to the reference library (Unigene database). Here the total tags represent total number of tags and the distinct tags represent total kinds of tags. The unambiguously mapped total tags, the tags map to only one gene, were 81.01% and 79.29%, and distinct tags were 60.85% and 65.67%. There were 6.48% and 7.46% unknown total tags, 19.76% and 15.36% of unknown distinct tags. Most of the unknown tags were expressed at relatively lower levels. Some of those genes expressed at low levels may represent background expression. Others may come from the incomplete documentation of expressed transcripts of the Unigene database (Table S1 and Figure S2).

The genes with expression copy number of less than 100 composed more than 50% (55.71% and 56.00%) of the total genes (Table 1). These genes may represent genes of background expression, non-specific expression, or transient expression. For effective analysis, we removed those genes in the following studies. About 11% (11.02% and 10.78%) genes have copy number of more than 500 copies.

**Table 1:** Gene expression levels classified by copy number.

Copy Number	Number of Genesin Dox+ sample	% of total genes	Number of genesin Dox− sample	% of total genes
2–99 copies	7550	55.71	7491	56.00
100–499 copies	4883	33.27	4876	33.22
≥500 copies	1617	11.02	1581	10.78

### Analysis of Uterus-expressed Genes

**Figure 2:**
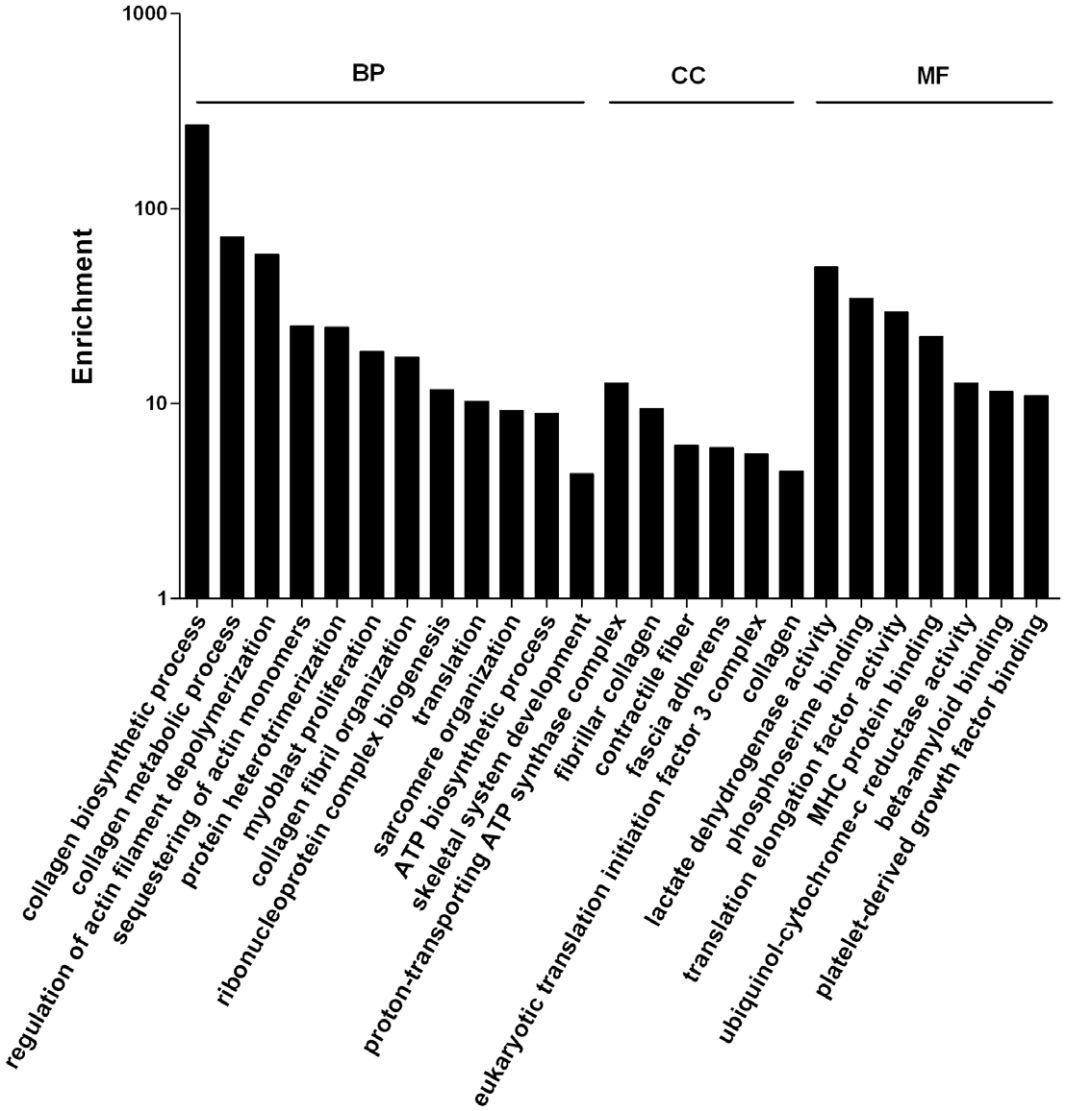
GO analysis of uterus-expressed genes. Genes with expression level higher than 100 copies were used. **BP** represents biological processes, **CC** represents cellular components and **MF** represents molecular functions.

For analysis of uterus-expressed genes, genes with 100 copies or more from Dox+ sample library were used. Dox+ sample was considered wild type alike. Total of 6500 genes were obtained after filtering. Figure 2 represents the result of the GO study. GO assignment was used to classify the function of genes, which can be presented into 3 categories, biological processes, cellular components and molecular functions. The major biological processes identified were collagen biosynthesis, collagen metabolic process, regulation of actin filament depolymerization, sequestering of actin monomers, protein heterotrimerization, myoblast proliferation and collagen fibril organization. The major cellular components were proton-transporting ATP synthase complex, fibrillar collagen, contractile fiber and fascia adherens. The molecular functions included lactate dehydrogenase activity, phosphoserine binding, translation elongation factor activities and MHC protein binding.

Further analysis indicated that genes with low copy numbers (100–499) were mostly coding for regulatory proteins involved in cell proliferation, transcriptional control and signal transductions (Figure S3). For example, the major biological processes were related to RNA splicing and GTPase activity, and the cellular component was Sin3 complex. Genes with high copy numbers (≥500) were mostly coding for structural, metabolizing and translational proteins (Figure S4). The most enriched bioprocesses were collagen biosynthesis, metabolism and organization. Genes involved in cellular components and molecular functions were ribosome and translation. Biological pathways were searched in KEGG database (www.genome.jp/kegg/). KEGG analysis indicated that the most enriched genes were involved in protein degradations and the most outstanding pathway was ribosome (Figure S5).

### Analysis of Uterus-specific Genes

**Figure 3:**
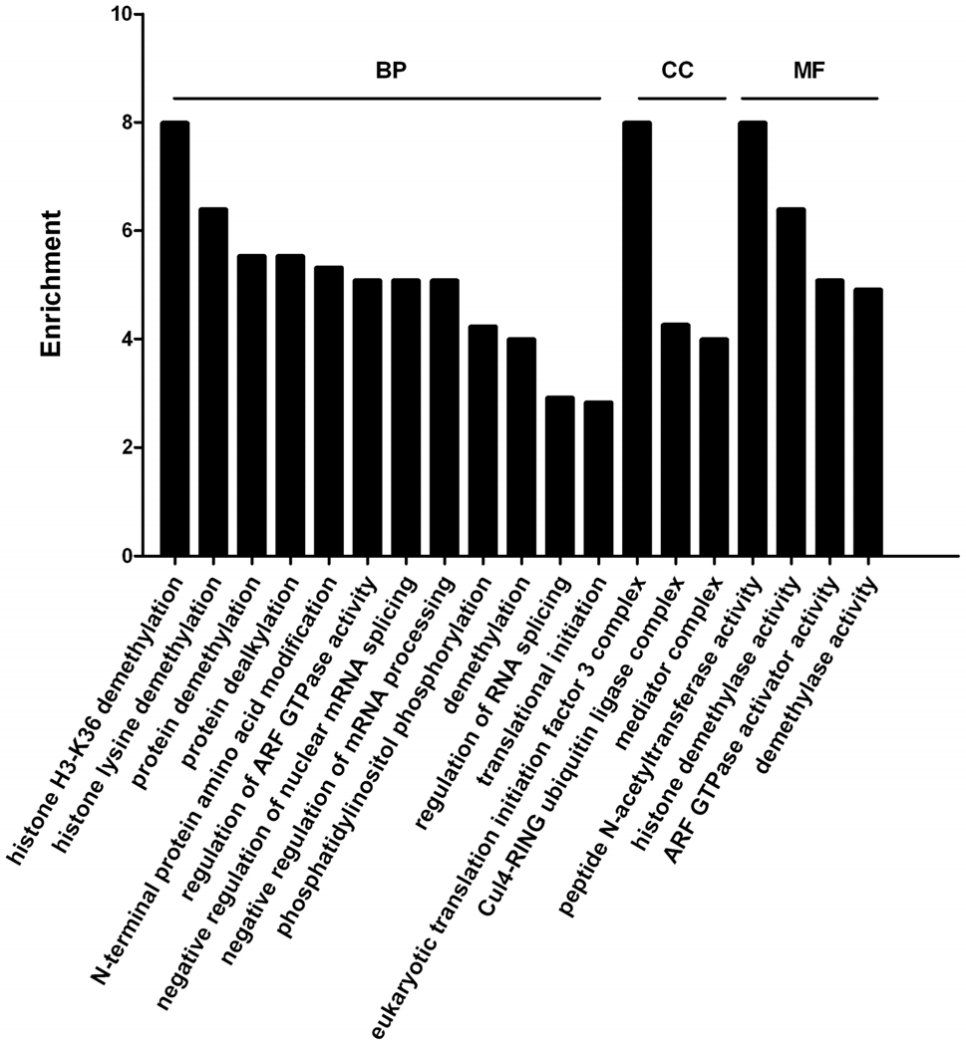
GO analysis of uterus-specific genes. Genes with expression level higher than 100 copies were used. Genes expressed in uterus were compared with genes in the liver, muscle and brain.

To analyze uterus-specific genes, genes with 100 copies or more from Dox+ sample library were used. Genes expressed in the uterus were compared with genes expressed in the liver, muscle and brain [27]. More than 828 uterus-specific genes were identified. GO and signaling pathways were analyzed against these genes. Figure 3 showed that histone demethylation, protein modifications and mRNA processing were the major biological processes. Translation initiation factor 3 complex and Cul4-RING ubiquitin ligase complex were the cellular components. Acetylation, demethylation and signaling kinases were among the active functional molecules. Results indicated that active histone modification and inositol metabolization might be specific and important during the pre-implantation stages. There were active spliceosome, endocytosis and inositol signaling found in the biologic pathways through KEGG analysis (Figure S6).

### Identification of VEGF-regulated Genes

**Figure 4:**
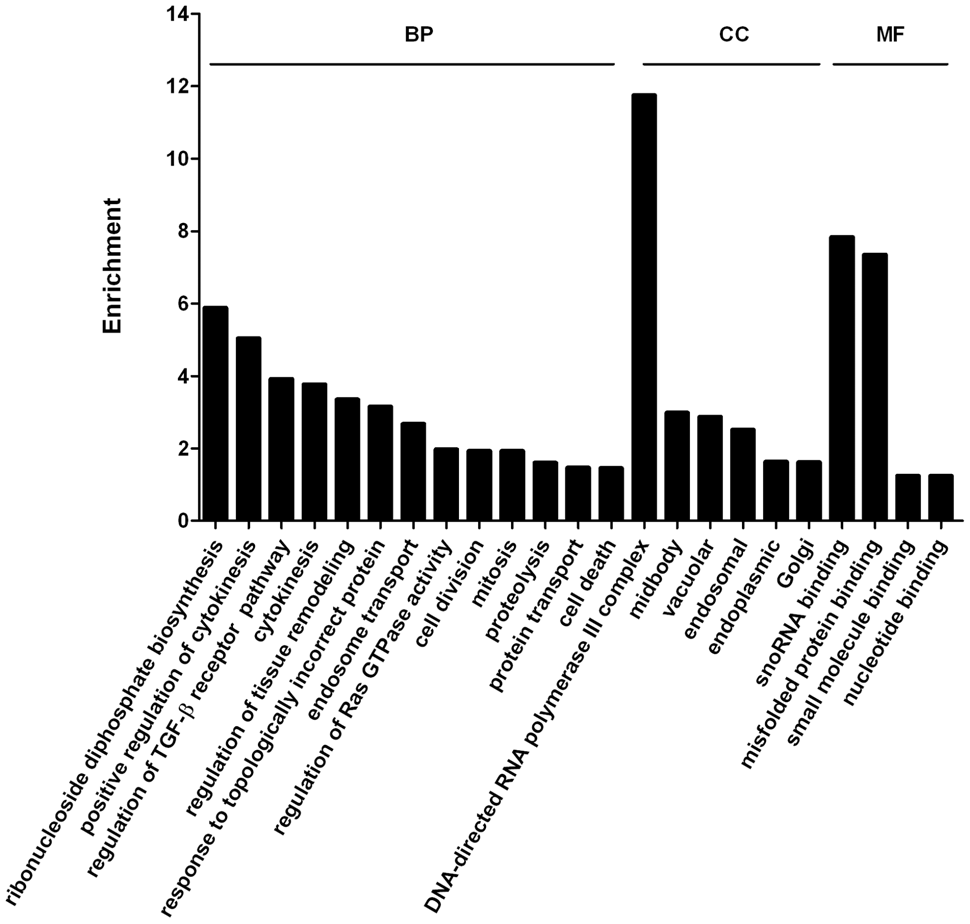
GO analysis of up-regulated genes by VEGF repression. Genes with combined expression level equal or higher than 200 copies (Dox+ plus Dox**−** ≥200) were used.

**Figure 5:**
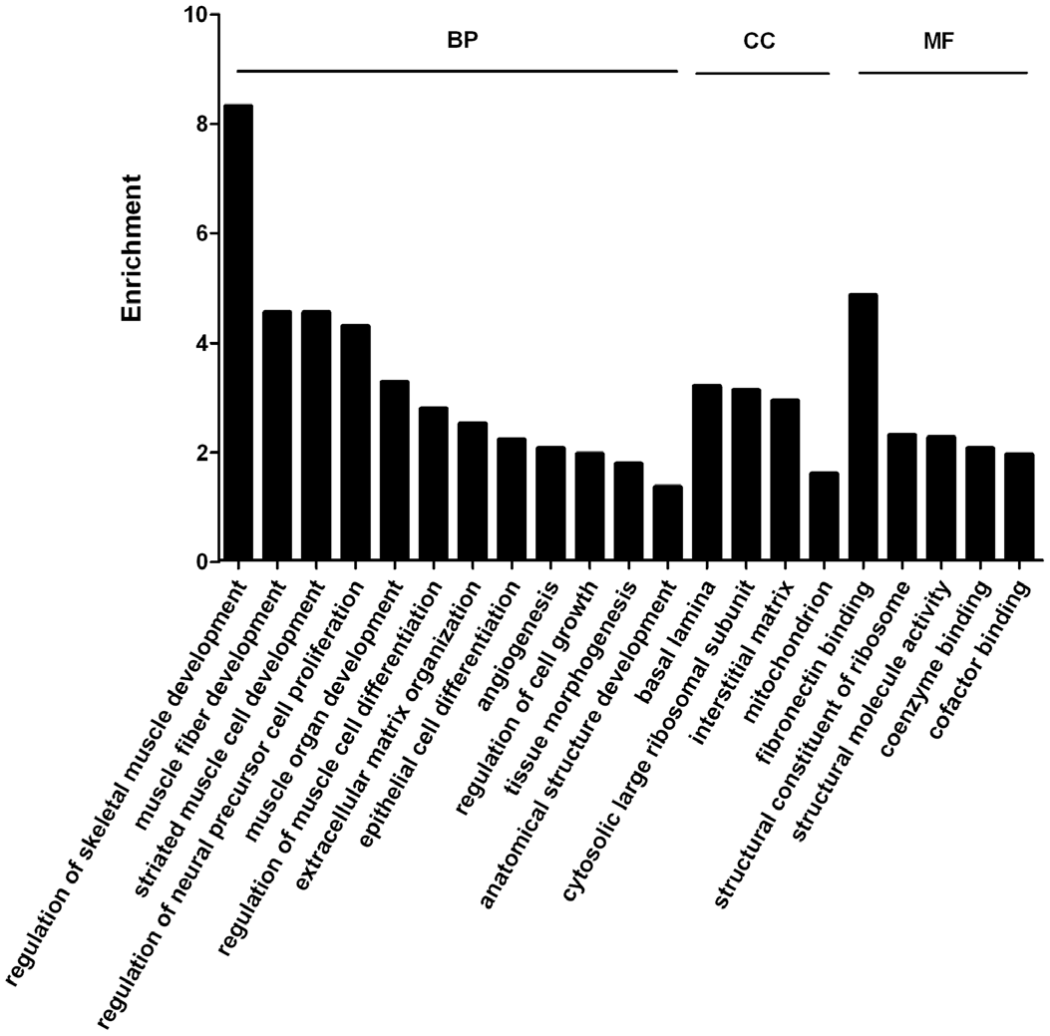
GO analysis of down-regulated genes by VEGF repression.

In order to study the mechanism of VEGF regulating implantation process and identify the genes that VEGF may regulate, the transcription profile of pre-implantation uterus was compared between Dox+ and Dox**−** samples. The cutoff of the combined copy number of each gene was ≥200 and minimum fold change was 1.5. A total of 2398 differentially expressed genes, 1231 up- and 1167 down-regulated, were identified. There were 302 up- and 368 down-regulated genes with fold change ≥2, 42 up- and 49 down-regulated genes with fold change ≥5 (Figure S7). Highly expressed and VEGF-regulated genes were listed in Table 2, Table S2 and Table S3. High percentages of up-regulated genes were found in the categories of ribonucleoside diphosphate biosynthetic process, cytokinesis, TGFβ pathway, regulation of tissue remodeling and RNA polymerase III function (Figure 4). Down-regulated genes were strongly associated with regulation of skeletal muscle tissue development, muscle fiber development, striated muscle cell development and fibronectin binding as shown in Figure 5. These results demonstrated VEGF might regulate muscle tissue remodeling. Previous studies have reported that TGFβ signaling pathway plays important roles in controlling apoptosis and cell survival during pregnancy [28].

**Table 2:** Top 20 VEGF*-*regulated genes.

Up-regulated Genes Symbol	Fold of Change	Down-regulated Genes Symbol	Fold of Change
keratin 85, Krt85	233.85	low density lipoprotein receptor-related protein 2,Lrp2	10000
inhibin beta-B, Inhbb	55.20	angiopoietin-like 7, Angpt17	58.824
cartilage acidic protein 1, Crtac1	52.36	sulfotransferase family 1D, member 1, Sult1d1	38.46
serine peptidase inhibitor, Kazal type 11, Spink12	50.68	N-acetylneuraminate pyruvate lyase, Np1	37.04
protein tyrosine phosphatase, non-receptor type 5, Ptpn5	41.61	junction adhesion molecule 2, Jam2	30.30
lipocalin 2, Lcn2	35.47	cytotoxic T lymphocyte-associated protein 2 alpha,Ctla2a	24.39
purinergic receptor P2Y, G-protein coupled, 14, P2ry14	35.05	lymphoid enhancer binding factor 1, Lef1	22.73
peptidyl arginine deiminase, type I, Padi1	26.93	Norrie disease (pseudoglioma), Ndp	18.87
major facilitator superfamily domain containing 4, Msfd4	23.01	monoamine oxidase B, Maob	17.54
protein phosphatase 1, regulatory (inhibitor) subunit 1B, Ppp1r1b	22.70	Indian hedgehog, Ihh	16.95
v-myc myelocytomatosis viral related oncogene, neuroblastoma derived, Mycn	21.86	FXYD domain-containing ion transport regulator 4,Fxyd4	16.95
gap junction protein, beta 2, Gjb2	16.47	follistatin, Fst	16.13
shisa homolog 2, Shisa2	16.38	histidine decarboxylase, Hdc	13.89
lecithin-retinol acyltransferase, Lrat2	12.97	metallothionein 2, Mt2	12.05
angel homolog 1, angel1	12.69	3-hydroxy-3-methylglutaryl-Coenzyme A synthase 2, Hmgcs2	11.63
sex comb on midleg-like 4, Scml4	11.53	HOXA11 antisense RNA, Hoxa11as	11.36
receptor (calcitonin) activity modifying protein 3, Ramp3	10.31	protein kinase domain containing, cytoplasmic, Pkdcc	10.64
cellular retinoic acid binding protein I, Crabp1	8.69	claudin 1, Cldn1	10.00
stratifin, Sfn	8.11	transcription factor 23, Tcf23	9.52
aldo-keto reductase family 1, member C14, Akr1c14	7.85	calbindin 1, Calb1	9.35

**Figure 6:**
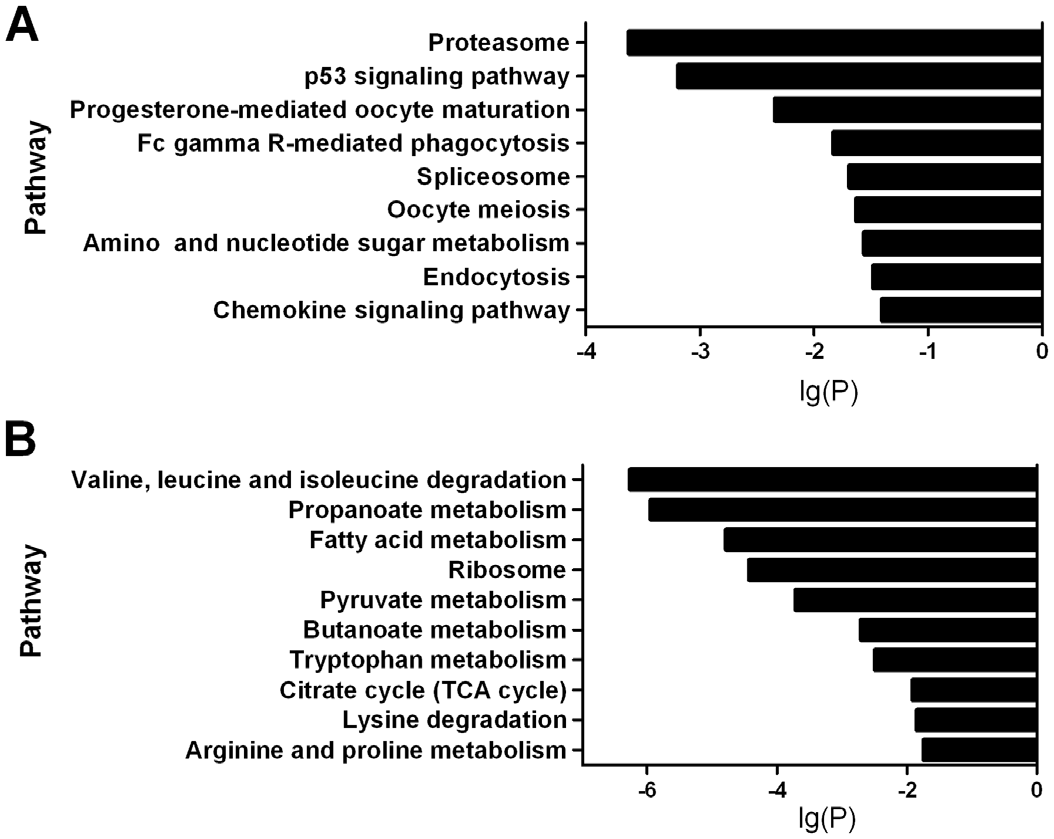
KEGG analysis of VEGF-regulated genes. Genes with expression level higher than 200 copies (Dox+ plus Dox**−** ≥200) were used. Pathway analysis was based on the Kyoto Encyclopedia of Genes and Genomes (KEGG) database. The Y-axis represents the pathway category and the X-axis represents the log10 (p-value) of the significant pathways.

KEGG analysis showed that up-regulated genes by VEGF repression were participating in proteasome, p53 signaling and progesterone-mediated oocyte maturation pathways (Figure 6A), indicating active protein degradation and apoptosis in developing uterus. Down-regulated genes were associated with valine, leucine, lysine degradation, propanoate, fatty acid, pyruvate metabolism and TCA cycle. Degradation and metabolic pathways were obviously down-regulated by VEGF repression (Figure 6B).

### Analysis of VEGF-regulated Antisense Transcript Expression

**Figure 7:**
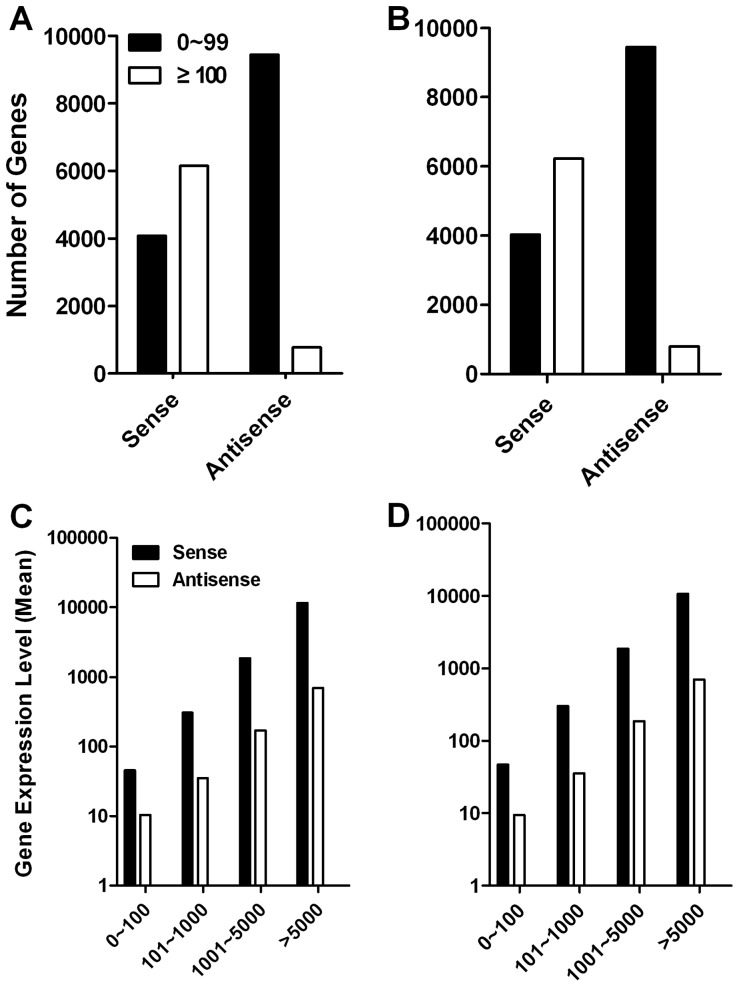
Antisense transcripts analysis. (**A**) Low and high expression sense and antisense transcript of Dox+ sample. (**B**) Low and high expression sense and antisense transcripts of Dox**−** sample. (**C**) Expression correlation analysis of sense and antisense transcripts in Dox+ sample (*r* = 0.9972). (**D**) Expression correlation analysis of sense and antisense transcripts in Dox**−** sample (*r* = 0.9968).

To determine whether VEGF regulates uterus development through antisense transcript expression, the antisense transcript levels were analyzed. There were 10247 and 10220 genes exhibited antisense transcriptions in Dox+ and Dox**−** uteri respectively. Most of antisense transcripts were less than 100 copies while sense expression was mostly more than 100 copies, indicating most of the antisense transcripts were probably non-specific (Figure 7A, 7B). Antisense expression levels showed correlation with sense expression, which also indicated possibility of non-specific transcription near active promoters and enhancers. The average expression level of antisense transcripts was about 10% of their sense transcripts (Figure 7C, 7D and Table S4). Analysis of antisense transcripts with copy number more than 100, 777 and 803 genes were expressed in Dox+ and Dox**−** samples, respectively. VEGF repression caused 310 up- and 205 down-regulation of antisense transcripts over 1.5 folds (Figure S8). The antisense transcripts with exceptionally high or low expression levels beyond the average were considered as important candidates in regulation of uterus development. Among them, 89 genes were up- and 80 down-regulated over 2.0 folds (Figure S8). Table S5 and Table S6 showed up- and down-regulated antisense transcripts over 3.0 folds.

### Validation of DGE Results by qPCR

**Figure 8:**
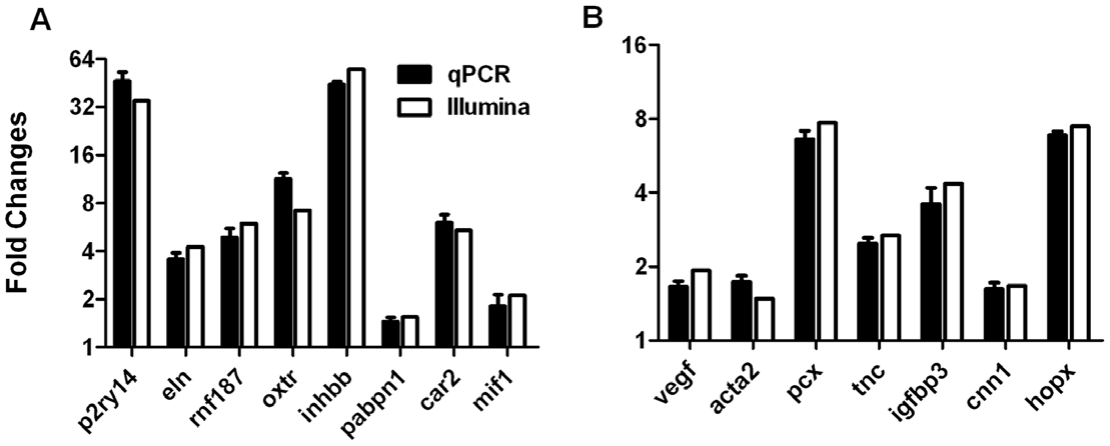
Validation of differentially expressed genes by qPCR. Representative genes were selected to validate the DGE results, (**A**) up-regulated genes and (**B**) down-regulated genes by VEGF repression. The Y-axis indicates the fold change of gene expression. Error bars represent standard error.

To validate the differentially expressed genes identified by DGE profiling, representative genes were confirmed by qPCR. The up-regulated genes included purinergic receptor P2Y, G-protein coupled, 14 (P2ry14), gap junction protein, beta 2 (Gjb2), oxytocin receptor (Oxtr) and carbonic anhydrase 2 (Car2) (Figure 8A). The down-regulated genes were VEGF, sulfotransferase family 1D, member 1 (Sult1d1), cytotoxic T lymphocyte-associated protein 2 alpha (Ctla2a), histidine decarboxylase (Hdc) and Indian hedgehog (Ihh) (Figure 8B). Pearson’s correlation coefficients (*r*) were 0.972 and 0.931 for up- and down-regulated genes respectively.

## Discussion

The critical role of VEGF in embryonic stage has been demonstrated by heterozygous lethality and dosage effect in the VEGF knockout mice [29]. It is difficult to study VEGF function in adult due to unavailable knockout mice and limited use of conditional knockout mice [30]. Our reversible repression system is an ideal model to overcome the above limits [19]. This study has shown that 54% reduction of VEGF mRNA had significant effect in number and scales of gene expression. More than 2000 genes were differentially expressed. Over 600 genes had more than 2 folds of change and near 100 genes more than 5 folds of change (Figure S7).

Among the highly expressed and VEGF-regulated genes, some have been previously identified active in uterus development and function. P2ry14, for example, is a G-protein coupled receptor for UDP-glucose, which triggers innate mucosal immunity in the female reproductive tract. It is exclusively expressed in human endometrial epithelial cells and plays important roles in preventing infection in the female reproductive tract. Patients with pelvic inflammatory disease showed increased IL8 and P2RY14 expression [31]. Our results suggest that VEGF may regulate P2ry14 expression but there is no indication of IL8 involvement as reported. Spink12, an orthologue of human LEKTI2, was expressed in the epidermis and several epithelia including uterus. It functions as an inhibitor against keratinocyte-derived trypsin-like protease in epithelial cells vital to maintain barrier function [32]. Lipocalin-2 (Lcn2) is implicated in diverse physiological processes including apoptosis, ion transport, inflammation, cell survival and tumorigenesis. It has been reported playing roles in the human female reproduction and endometrial cancer [33]. Our study also identified Inhibin beta-B (Inhbb) which is subunit of activin and inhibin, two closely related glycoproteins but playing opposite biological roles [34]. Inhibin and activin family has been found playing roles in the paracrine regulation of endometrial receptivity, decidualization and implantation [35]. Protein tyrosine phosphatase non-receptor type 5 (Ptpn5) is highly expressed in muscle, uterus, skin and uterine corpus endometrioid carcinoma [36]. All the above genes were highly expressed and regulated by VEGF repression, but their connective roles in uterus preparation for implantation are still missing.

Some other highly expressed and regulated genes are known for their expression in uterus but roles in uterus function are not clear. Keratin 85 (Krt85) is a member of the keratin family expressed in muscle and uterus. It codes a type II hair keratin, a basic protein that heterodimerizes with type I keratins to form hair and nails [37]. Sulfotransferase family 1D, member 1 (Sult1d1) is another uterus and muscle expressed gene. FXYD domain-containing ion transport regulator 4 (Fxyd4) is a family member of small membrane proteins expressed in vascular smooth muscle [38], [39] and Metallothionein 2 (Mt2) is a metal binding protein and known to be involved in many functions including muscle contraction [40], [41]. These genes were down-regulated by VEGF repression and their roles need to be reinvestigated.

There are many genes expressed and up-regulated by VEGF repression in endometrium. CD24, for example, has been found expressed in a cyclic pattern in the normal endometrium and endometrial carcinoma [42]. The oxytocin receptor (Oxtr), receptor for the hormone and neurotransmitter oxytocin, has been found expressed in both myometrium and endometrium of uterus [43], [44]. ADH1 is specifically expressed in endometrial stromal cells, up-regulated by progesterone and may regulate retinoic acid synthesis [45].

Some genes are identified and found playing important function in other tissues but have never been reported in uterus. Gap junction beta-2 protein (Gjb2) is composed of two hemichannels and each has six connexin molecules. The structural feature of Gjb2 allows coupling of electrical and biochemical signals between cells and tissues, and generation of synchronized responses. Mutations of Gjb2 are associated with sensorineural deafness, skin disorders, peripheral neuropathy and cardiovascular disease [46], [47]. However, its expression is limited to brain and its roles in uterine epithelial cells have never been reported.

There are still other genes, including angel homolog 1 (Angel1), e74-like factor 3 (Elf3), ring finger protein 187 (Rnf187), receptor activity modifying protein 3 (Ramp3), carbonic anhydrase 2 (Car2) and collagen, type VI, alpha 4 (Col6a4), were highly expressed and up-regulated but with very limited information. Calbindin 1 (Calb1), protein kinase domain containing-cytoplasmic (Pkdcc), glutathione S-transferase, mu 1 (Gstm1) and cytotoxic T lymphocyte-associated protein 2 alpha (Ctla2a) were highly expressed and down-regulated by VEGF repression. These genes are found first time in uterus and their potential biologic functions should be further investigated.

Estrogen and progesterone are basic steroid hormones for reproduction. Their related synthases and receptors have been widely studied in the past decades [48]. We have found more than dozen of genes that are responsive to estrogen stimulation (Table 3). Oxtr, for example, is up-regulated more than 7 folds and Indian hedgehog (Ihh) is down-regulated more than 17 folds.

**Table 3:** VEGF-regulated genes associated to estrogen stimulus.

Up-regulated Gene Symbol	Fold of Change	Down-regulated Gene Symbol	Fold of Change
oxytocin receptor, Oxtr	7.23	Indian hedgehog, Ihh	17.05
peptidylglycine alpha-amidating monooxygenase, Pam	3.61	WAP four-disulfide core domain 1, Wfdc1	3.58
aldehyde dehydrogenase family 1, subfamily A2,Aldh1a2	2.77	annexin A1, Anxa1	2.41
cyclin A2, Ccna2	2.02	thioredoxin interacting protein, Txnip	2.18
matrix metallopeptidase 15, Mmp15	1.93	crystallin, alpha B, Cryab	1.72
suppressor of cytokine signaling 3, Socs3	1.66	phosphatase and tensin homolog, Pten	1.52
estrogen receptor 1, Esr1	1.63	nuclear receptor subfamily 2, group F,member 2, Nr2f2	1.50
suppressor of cytokine signaling 2, Socs2	1.60		
caspase 8, Casp8	1.52		

Among the uterus-expressed genes, many genes that were expressed at lower level have been functionally associated with regulation of cell proliferation and cell signaling. When analyzing genes expressed at higher levels, most of the genes were found to be participants in cellular structure and metabolism. The most striking group of genes, which are down-regulated by VEGF repression, are associated with muscle development and function (Figure 5). Over 800 uterus-specific genes were identified. Among them, one of the major groups was involved in histone modifications, an indication of active epigenetic regulation in uterus. Many genes, for example, are participating in histone H3-K36 demethylation and histone lysine demethylation. H3-K36 trimethylation has been found associated with active transcription.

Antisense transcripts have been found, in many cases, playing critical roles in developmental regulation [49]. Surprisingly, antisense expression is unexpectedly high and correlated to the sense expression. Most of antisense expressions at low levels may be non-specific and probably generated from the active promoters and enhancers. Nevertheless, there were antisense transcripts beyond the average, up- or down-regulated by VEGF repression. Those transcripts expressed at very high or very low levels under VEGF regulation is worth of further investigation. Hox, for example, plays important roles in Wnt signaling during uterine development [50], [51], [52]. Its expression is regulated by progesterone and menstrual cycles. Hoxa11 antisense RNA (Hoxa11 as) was highly expressed and down-regulated by VEGF repression. The antisense transcripts may down-regulate Hox11 gene expression [53]. NME/NM23 family member 7 (Nme7) has been reported to be widely expressed in organs including uterus, a protein that exhibits ATP binding, metal ion binding and nucleoside diphosphate kinase activity. Its function involves brain development, smoothened signaling pathway and determination of left/right symmetry [54]. Nme7 antisense transcript was expressed at much higher level than sense transcript and regulated by VEGF. Other important antisense transcripts include four and a half LIM domains 1 (Fhl1) and CD24a antigen (Cd24a) that were also highly expressed and regulated by VEGF repression. The importance of these antisense transcripts needs to be determined.

In summary, this study has used the reversible repression system to study VEGF effect on uterus gene expression during pre-implantation stage. Many genes were found to be regulated by VEGF. VEGF participates in many biological processes and various functions, and probably regulates gene expression at higher levels. This study will provide large amount of information for researchers in the field. Further study is needed to discover their localizations and relationships to uterus development during the implantation stage.

## Supporting Information

Figure S1 **Saturation analysis of Dox+ and Dox− tag libraries.** The number of detected genes increased with the amount of data output. When the library size reached 1 million, approximate of 60% and 56% of documented genes were identified respectively, which were close to saturation in a particular tissue.
(TIF)

Figure S2 **Tag distribution.** Number in the square brackets indicated specific category by copy number. Number in the parentheses indicated total numbers and percentage of tags in the category. Genes expressed with 100 copies or more took most of tags (76.80% to 74.03%), while genes expressed with 2–5 copies took most genes (64.56% to 61.99%), which may indicate background expression.
(TIF)

Figure S3 **GO analysis of uterus-expressed genes with copy number between 100–499.**
(TIF)

Figure S4 **GO analysis of uterus-expressed genes with copy number more than 500.**
(TIF)

Figure S5 **KEGG analysis of uterus-expressed genes.** Genes with expression level higher than 100 copies were used. Signaling pathways which had a P-value<0.05 and FDR<0.05 were considered significant.
(TIF)

Figure S6 **KEGG analysis of uterus-specific genes.**
(TIF)

Figure S7 **Range distribution of up- and down-regulated genes.** Genes with combined expression level equal or higher than 200 copies (Dox+ plus Dox**−** ≥200) were used. There were 1231 up- and 1167 down-regulated genes with fold change ≥1.5 (**A**), 302 up- and 368 down-regulated genes with fold change ≥2.0 (**B**), and 42 up- and 49 down-regulated genes with fold change ≥5.0 (**C**), respectively.
(TIF)

Figure S8 **Range distribution of up- and down-regulated antisense transcripts.** There were 310 up- and 205 down-regulated antisense transcripts with fold change ≥1.5 (**A**), 89 up- and 80 down-regulated antisense transcripts with fold change ≥2.0 (**B**), and 6 up- and 11 down-regulated antisense transcripts with fold change ≥5.0 (**C**), respectively.
(TIF)

Table S1 **Total tags represented total number of clean tags, while distinct tags represented total kinds of tags.** Unambiguous mapped tags indicated the tags matched to only one gene.
(DOC)

Table S2 **Up-regulated genes by VEGF repression at G2.5.** The table showed the most highly expressed and VEGF-regulated genes with fold change ≥5.0.
(XLS)

Table S3 **Down-regulated genes by VEGF repression at G2.5.** The most highly expressed and VEGF-regulated genes with fold change ≥5.0.
(XLS)

Table S4 **Analysis of antisense transcript expression.**
(DOC)

Table S5 **VEGF up-regulated antisense transcripts.** Table showed fold of change over 3.0 and expression level more than 100 copies.
(XLS)

Table S6 **VEGF down-regulated antisense transcripts.** Table showed fold of change over 3.0 and expression level more than 100 copies.
(XLS)

Table S7 **Primer sequences for qPCR.**
(XLS)
