# Supplementary figures and images for: Transcriptional Profiling of Mouse Uterus at Pre-Implantation Stage under VEGF Repression
Source: PLoS One. 2013 Feb 28;8(2):e57287. doi: 10.1371/journal.pone.0057287 (PMC3585347; doi:10.1371/journal.pone.0057287)

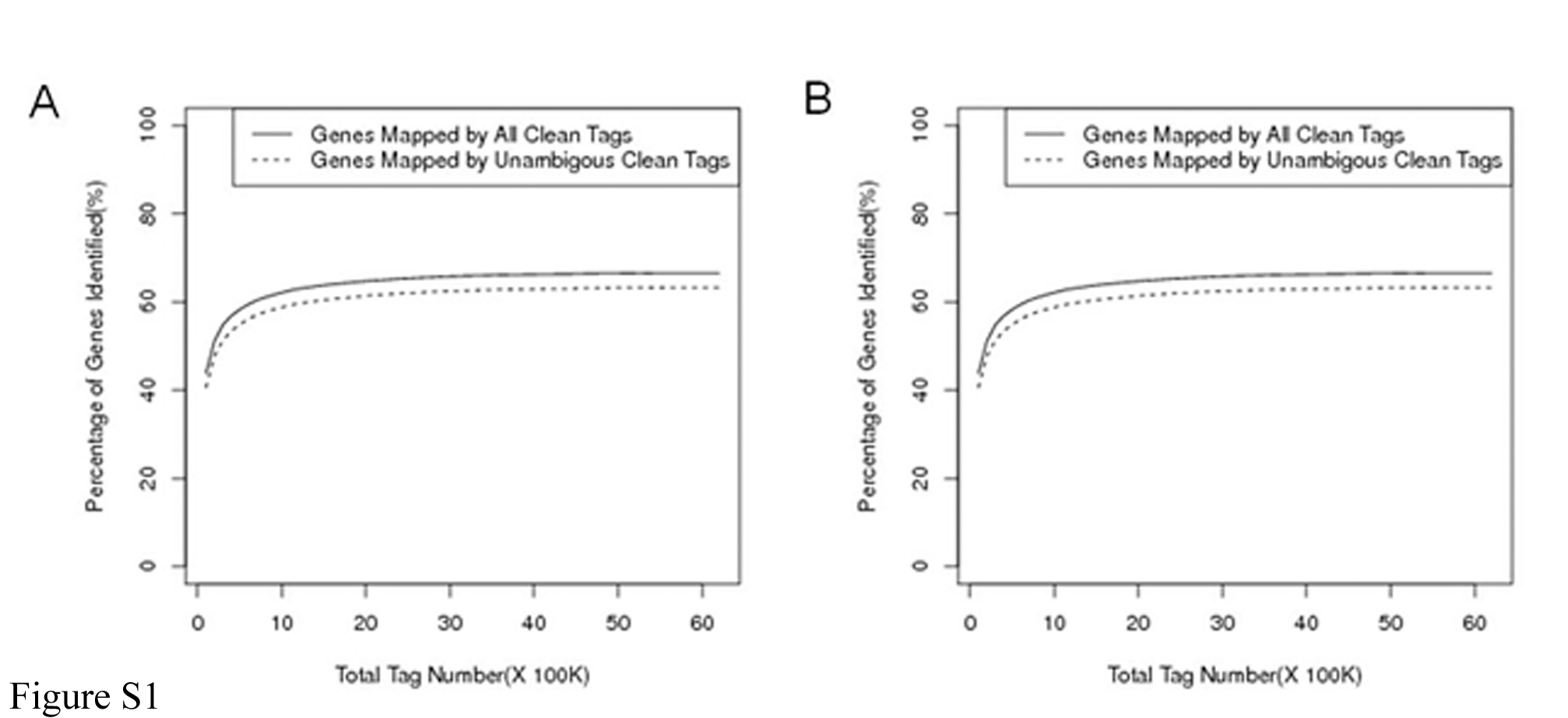

Supplement: Figure S1 — Saturation analysis of Dox+ and Dox− tag libraries. The number of detected genes increased with the amount of data output. When the library size reached 1 million, approximate of 60% and 56% of documented genes were identified respectively, which were close to saturation in a particular tissue. (TIF) [file pone.0057287.s001.tif]

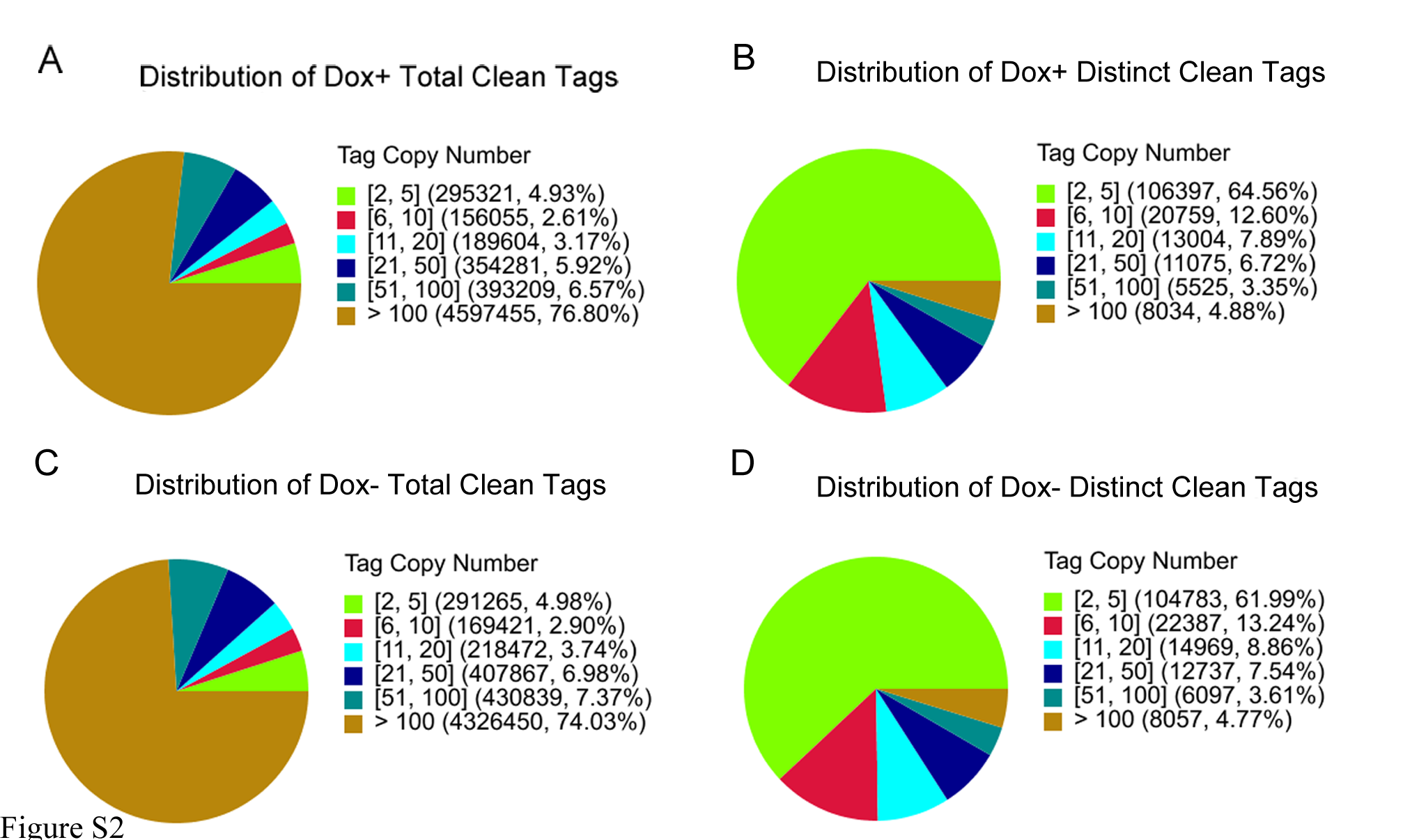

Supplement: Figure S2 — Tag distribution. Number in the square brackets indicated specific category by copy number. Number in the parentheses indicated total numbers and percentage of tags in the category. Genes expressed with 100 copies or more took most of tags (76.80% to 74.03%), while genes expressed with 2–5 copies took most genes (64.56% to 61.99%), which may indicate background expression. (TIF) [file pone.0057287.s002.tif]

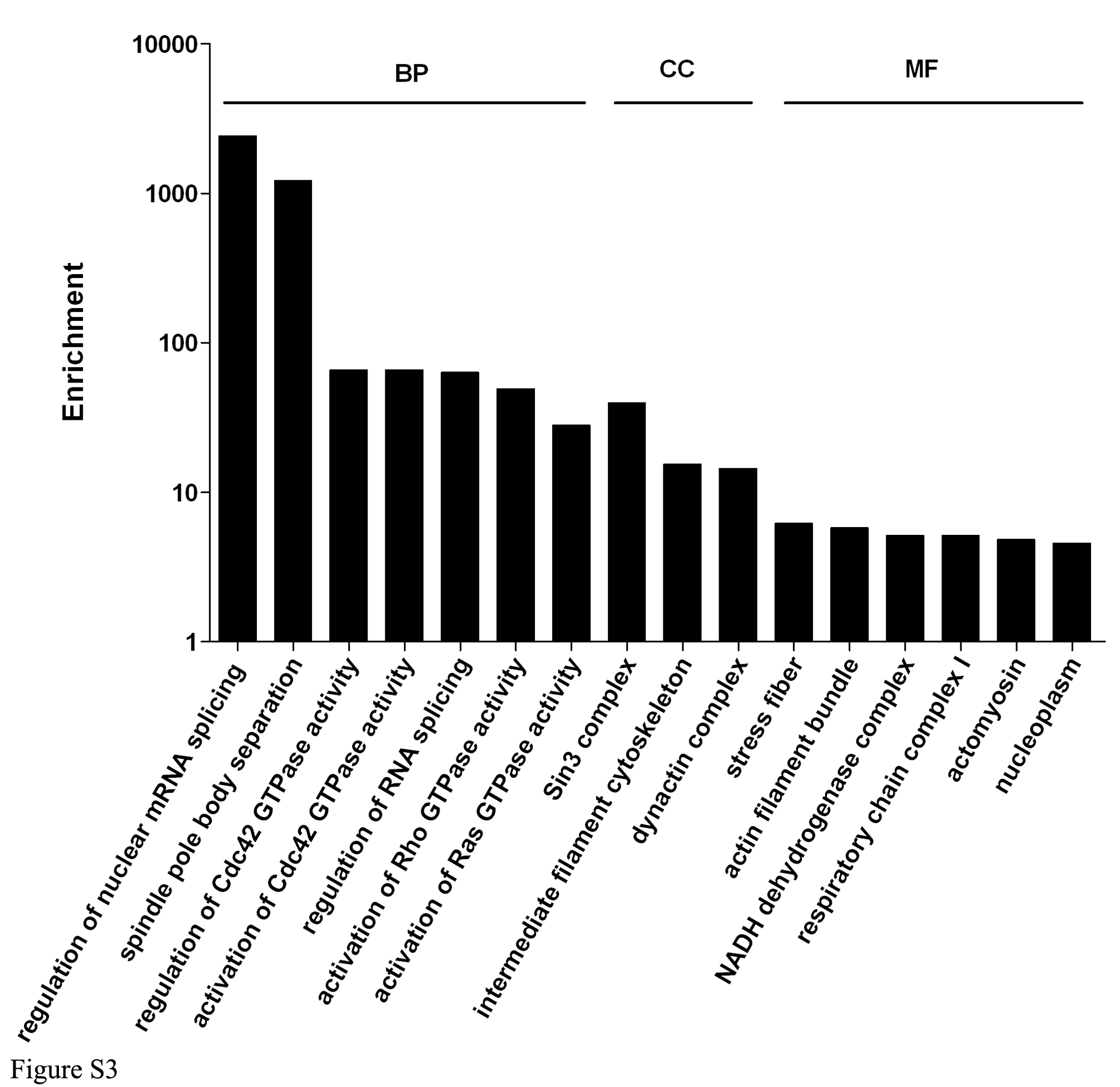

Supplement: Figure S3 — GO analysis of uterus-expressed genes with copy number between 100–499. (TIF) [file pone.0057287.s003.tif]

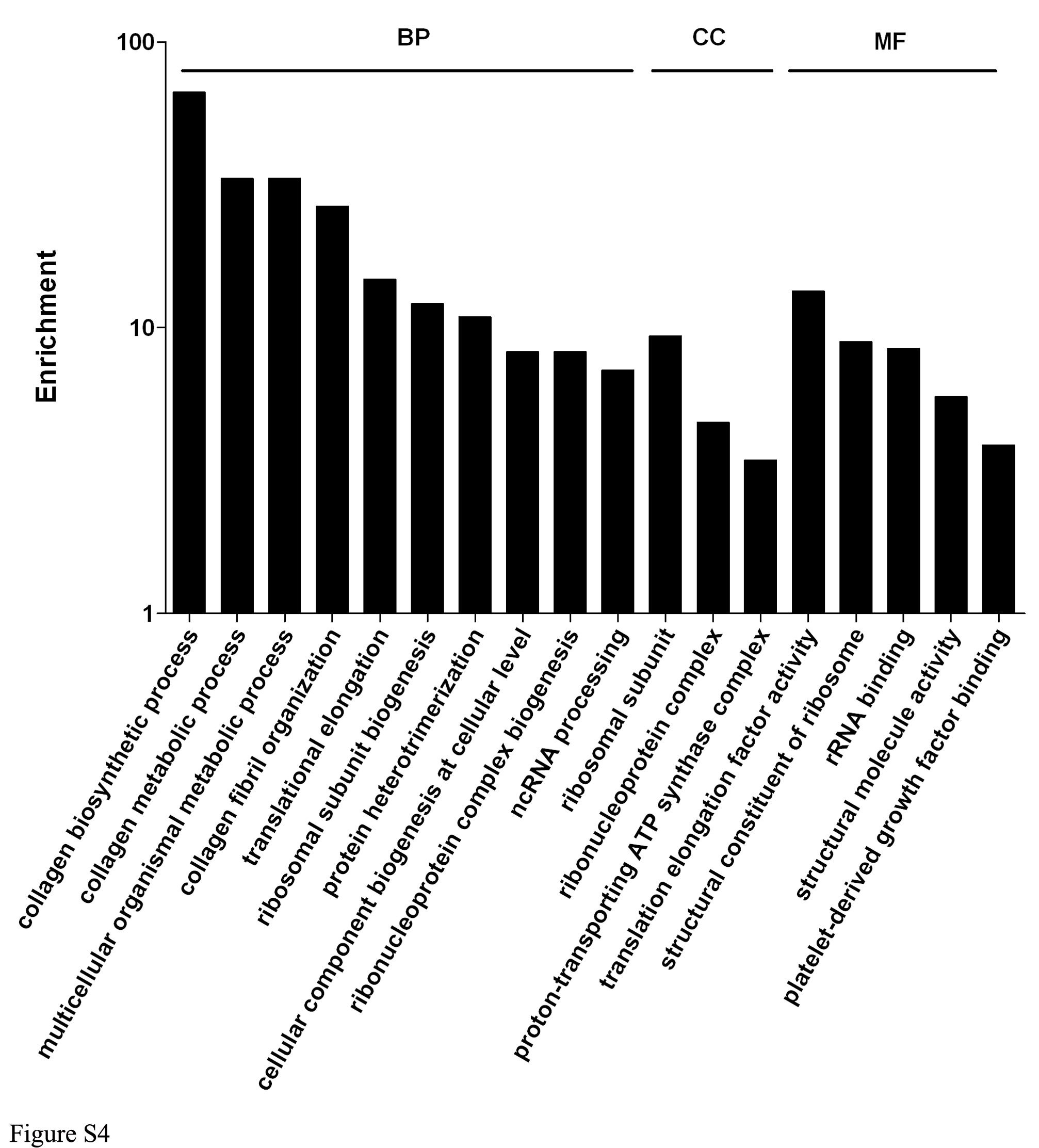

Supplement: Figure S4 — GO analysis of uterus-expressed genes with copy number more than 500. (TIF) [file pone.0057287.s004.tif]

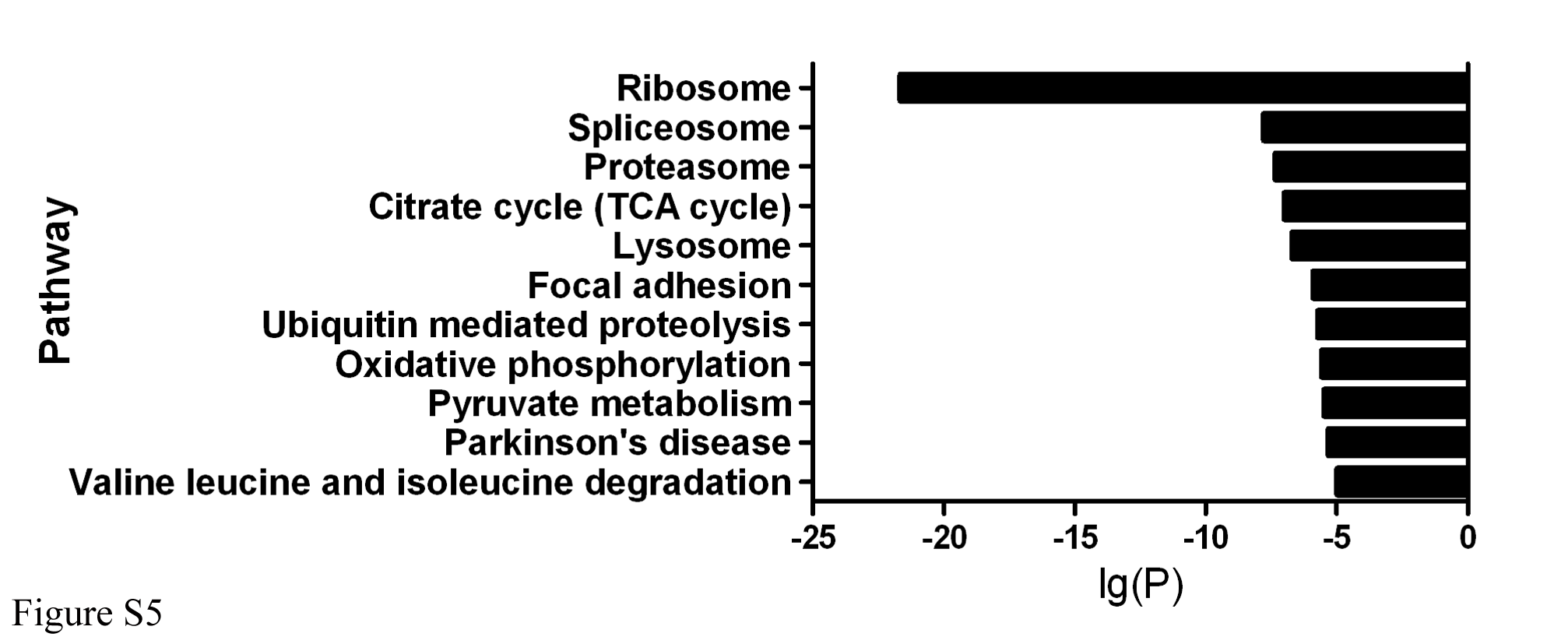

Supplement: Figure S5 — KEGG analysis of uterus-expressed genes. Genes with expression level higher than 100 copies were used. Signaling pathways which had a P-value<0.05 and FDR<0.05 were considered significant. (TIF) [file pone.0057287.s005.tif]

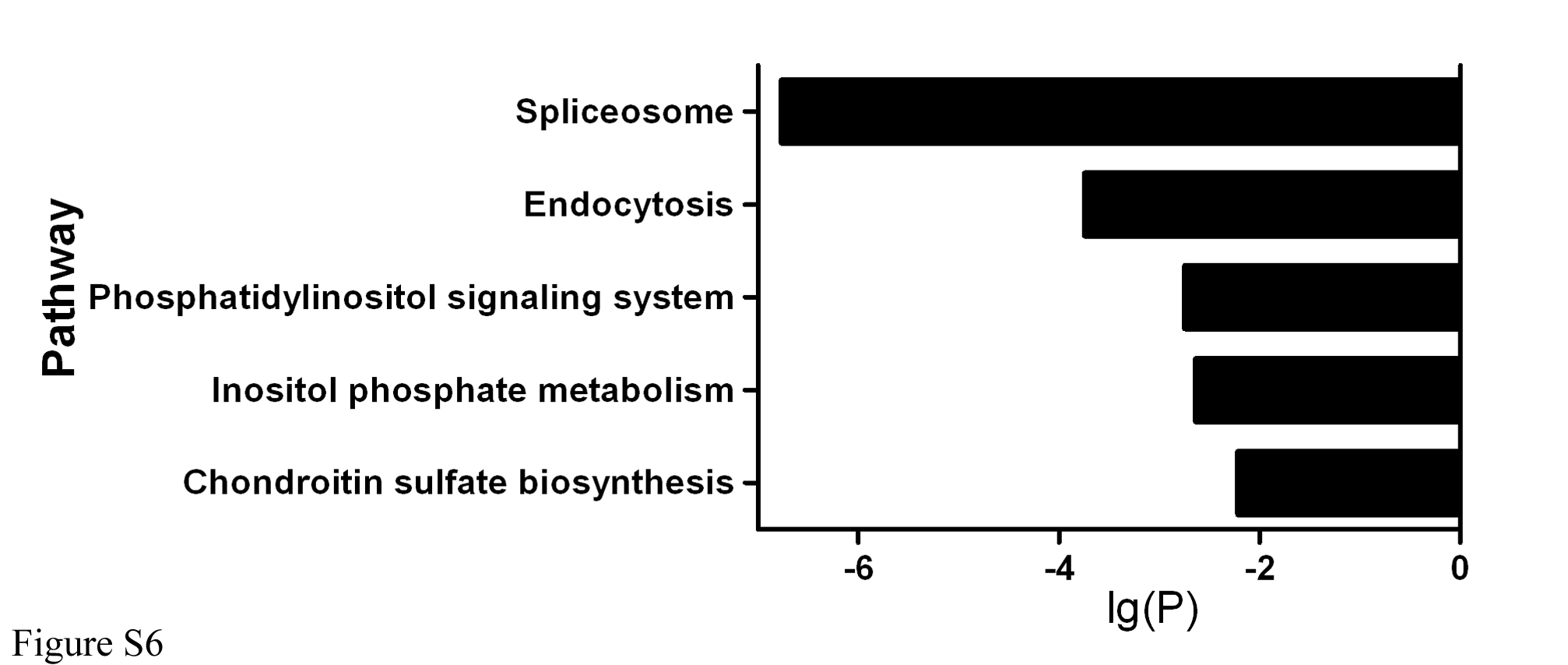

Supplement: Figure S6 — KEGG analysis of uterus-specific genes. (TIF) [file pone.0057287.s006.tif]

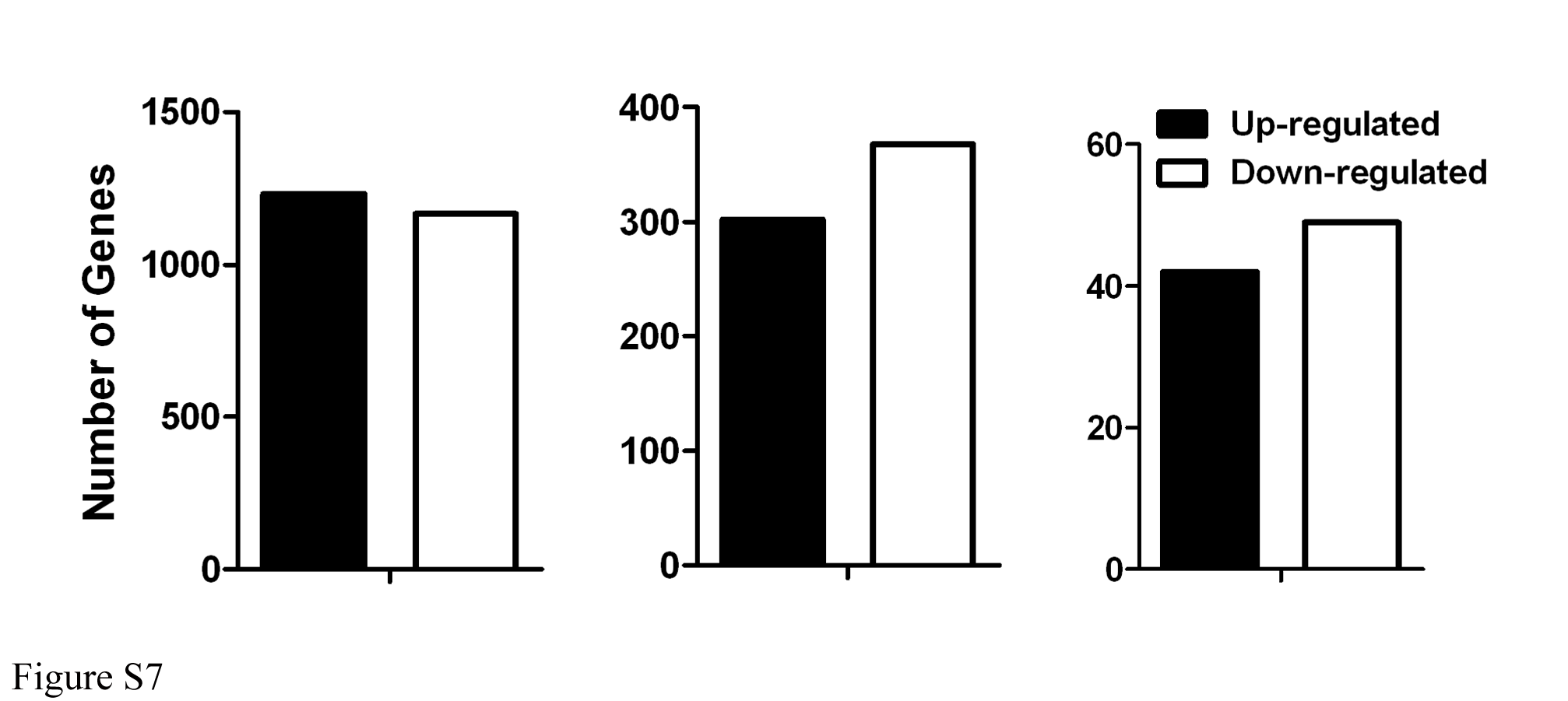

Supplement: Figure S7 — Range distribution of up- and down-regulated genes. Genes with combined expression level equal or higher than 200 copies (Dox+ plus Dox− ≥200) were used. There were 1231 up- and 1167 down-regulated genes with fold change ≥1.5 (A), 302 up- and 368 down-regulated genes with fold change ≥2.0 (B), and 42 up- and 49 down-regulated genes with fold change ≥5.0 (C), respectively. (TIF) [file pone.0057287.s007.tif]

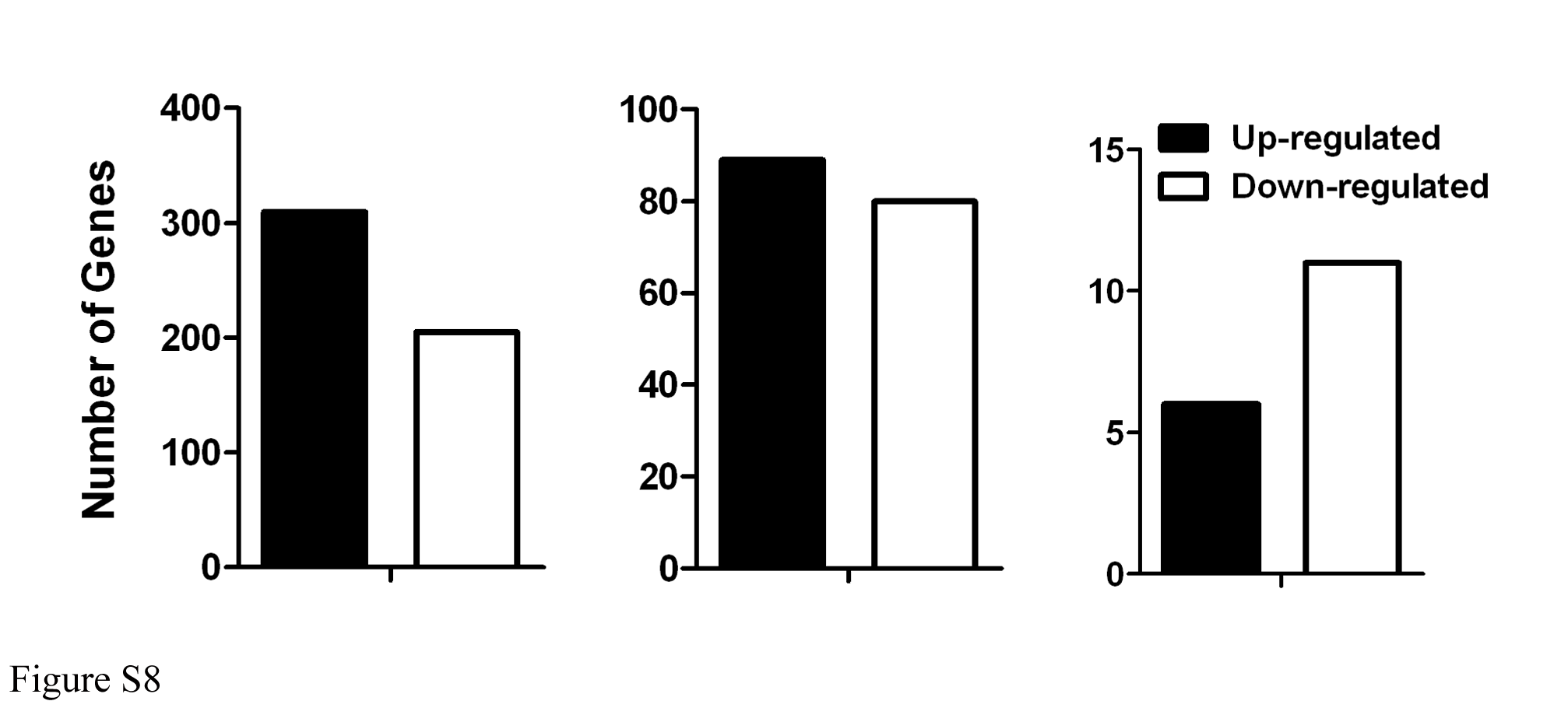

Supplement: Figure S8 — Range distribution of up- and down-regulated antisense transcripts. There were 310 up- and 205 down-regulated antisense transcripts with fold change ≥1.5 (A), 89 up- and 80 down-regulated antisense transcripts with fold change ≥2.0 (B), and 6 up- and 11 down-regulated antisense transcripts with fold change ≥5.0 (C), respectively. (TIF) [file pone.0057287.s008.tif]
