# Supplementary material for: Transcriptional Profiling of Mouse Uterus at Pre-Implantation Stage under VEGF Repression
Source: PLoS One. 2013 Feb 28;8(2):e57287. doi: 10.1371/journal.pone.0057287 (PMC3585347; doi:10.1371/journal.pone.0057287)
Supplement: Table S1 — Total tags represented total number of clean tags, while distinct tags represented total kinds of tags. Unambiguous mapped tags indicated the tags matched to only one gene. (DOC) [file pone.0057287.s009.doc]

| **Tags** | **Dox+** | | **Dox-** | |
| --- | --- | --- | --- | --- |
|  | **Total tags** | **Distinct Tags** | **Total tags** | **Distinct Tags** |
| Raw Tags | 6143402 | 321211 | 6025112 | 348766 |
| Clean tags  (% to raw tags) | 5985925 (97.44%) | 164794  (51.30%) | 5844314  (97.00%) | 169030  (48.47%) |
| Copy Number >100  (% to clean tags) | 4597455  (76.80%) | 6500  (3.94%) | 4326450  (74.03%) | 6458  (3.82%) |
| Mapped clean tags  (% to clean tags) | 5205527  (86.96%) | 103642  (62.89%) | 4987573  (85.34%) | 114602  (67.80%) |
| Unambiguous mapped Tags  (% to clean tags) | 4849001  (81.01%) | 100282  (60.85%) | 4633643  (79.29%) | 111006  (65.67%) |
| Unknown Tags  (% to clean tags) | 386782  (6.46%) | 32561  (19.76%) | 436062  (7.46%) | 25958  (15.36%) |
