# Supplementary material for: Transcriptional Profiling of Mouse Uterus at Pre-Implantation Stage under VEGF Repression
Source: PLoS One. 2013 Feb 28;8(2):e57287. doi: 10.1371/journal.pone.0057287 (PMC3585347; doi:10.1371/journal.pone.0057287)
Supplement: Table S4 — Analysis of antisense transcript expression. (DOC) [file pone.0057287.s012.doc]

| **Numbers of genes at specific expression levels** | | | | |
| --- | --- | --- | --- | --- |
|  | Dox+ | | Dox- | |
| Expression levels | 0~99 | ≥100 | 0~99 | ≥100 |
| Sense | 4074 | 6147 | 4026 | 6222 |
| Antisense | 9443 | 778 | 9445 | 803 |
| **Average expression levels in a particular range** | | | | |
| Expression ranges | Sense | Antisense | Sense | Antisense |
| 0~100 | 45.542 | 10.373 | 47.043 | 9.424 |
| 101~1000 | 306.635 | 35.231 | 302.023 | 35.526 |
| 1001~5000 | 1855.616 | 170.828 | 1861.928 | 185.425 |
| >5000 | 11600.73 | 696.159 | 10633.81 | 701.019 |
| **Percentage of antisense to sense transcript in a particular range** | | | | |
| Expression ranges | Dox+ | | Dox- | |
| 0-100 | 22.78% | | 20.03% | |
| 101-1000 | 11.49% | | 11.76% | |
| 1000-5000 | 9.21% | | 9.94% | |
| >5000 | 6.00% | | 6.6% | |
| Average≥100 | 8.9% | | 9.43% | |
